# Treatment topical with silver nanoparticles and *Stryphnodendron Adstringens* (Mart.) *Coville* in cervical lesions: randomized clinical trial

**DOI:** 10.1590/1980-220X-REEUSP-2022-0338en

**Published:** 2023-07-28

**Authors:** Cristiane Araújo Nascimento, Ana Caroline Melo dos Santos, Denise Macedo da Silva, Nirliane Ribeiro Barbosa, Edilson Leite de Moura, Veracilda Bezerra da Silva, Tatiane Luciano Balliano, Elaine Virginia Martins de Souza Figueiredo, Karol Fireman de Farias, Guilherme Benjamin Brandão Pitta

**Affiliations:** 1 Universidade Federal de Alagoas, Arapiraca, AL, Brazil.; 2 Hospital de Emergência Dr. Daniel Houly, Arapiraca, AL, Brazil.; 3 Universidade Federal de Alagoas, Programa de Pós-Graduação em Ciências da Saúde, Maceió, AL, Brazil.; 4 Universidade Federal de Pernambuco, Programa de Pós-Graduação em Biotecnologia em Saúde, Recife, PE, Brazil.; 5 Farmácia de Manipulação Pharmavida, Arapiraca, AL, Brazil.; 6 Universidade Federal de Alagoas, Instituto de Química e Biotecnologia, Maceió, AL, Brazil.; 7 Universidade Federal de Alagoas, Laboratório de Biologia Molecular e Expressão Gênica, Programa de Pós-Graduação em Enfermagem, Arapiraca, AL, Brazil.; 8 Universidade Federal de Alagoas, Laboratório de Inovação e Biotecnologia em Saúde, Maceió, AL, Brazil.; 9 Universidade Estadual de Ciências da Saúde de Alagoas, Programa de Pós-Graduação em Biotecnologia em Saúde, Maceió, AL, Brazil.

**Keywords:** Squamous Intraepithelial Lesions of the Cervix, Uterine Cervical Neoplasms, Stryphnodendron barbatimam, Phytotherapy, Lesões Intraepiteliais Escamosas Cervicais, Neoplasias do Colo do Útero, Stryphnodendron barbatimam, Fitoterapia, Lesiones Intraepiteliales Escamosas de Cuello Uterino, Neoplasias del Cuello Uterino, Stryphnodendron barbatimam, Fitoterapia

## Abstract

**Objective:**

To assess the feasibility of incorporating technology as a new alternative for treating topics on cervical lesions.

**Method:**

This is a randomized, double-blind, controlled clinical trial with a prospective design. During the realization of this study, 43 women were included and divided between groups A (ointment without silver nanoparticles n = 23) and B (ointment with silver nanoparticles n = 20) clinically healthy and who used the unified health system.

**Results:**

There were no significant differences when comparing before and after the use of ointment for IVA test (p = 0.15), Schiller test (p = 0.15), cellular changes (p = 0.47) and microbiological analysis (p = 0.89) through cytology. After use, no adverse reaction was observed in the sample studied.

**Conclusion:**

Based on the results identified in this study, identified that the product is safe and does not promote adverse events. Regarding the effectiveness of the product in uterine cervical lesions, it is necessary to continue the study in phase II. Registro de Ensaios Clínicos Brasileiros: UTN: U1111-1218-2820.

## RESUMO

**Objetivo:**

Avaliar a viabilidade da incorporação da tecnologia como uma nova alternativa para o tratamento de lesões cervicais.

**Método:**

Trata-se de um ensaio clínico randomizado, duplo-cego e controlado com um desenho prospectivo. Durante a realização deste estudo, foram incluídas 43 mulheres, divididas entre os grupos A (pomada sem nanopartículas de prata n = 23) e B (pomada com nanopartículas de prata n = 20), clinicamente saudáveis e usuárias do sistema único de saúde.

**Resultados:**

Não houve diferenças significativas na comparação entre antes e depois do uso da pomada para o teste IVA (p = 0,15), teste de Schiller (p = 0,15), alterações celulares (p = 0,47) e análise microbiológica (p = 0,89) por meio de citologia. Após o uso, não foi observada nenhuma reação adversa na amostra estudada.

**Conclusões:**

Com base nos resultados identificados neste estudo, identificou-se que o produto é seguro e não promove eventos adversos. Com relação à eficácia do produto em lesões cervicais uterinas, é necessária a continuidade do estudo na fase II. Registro de Ensaios Clínicos Brasileiros: UTN: U1111-1218-2820.

## RESUMEN

**Objetivo:**

evaluar la viabilidad de la incorporación de la tecnología como una nueva alternativa para el tratamiento de temas sobre lesiones cervicales.

**Método:**

Se trata de un ensayo clínico aleatorizado, doble ciego, controlado y con un diseño prospectivo. Durante la realización de este estudio se incluyeron 43 mujeres divididas entre los grupos A (pomada sin nanopartículas de plata n = 23) y B (pomada con nanopartículas de plata n = 20) clínicamente sanas y usuarias del sistema unificado de salud.

**Resultados:**

No hubo diferencias significativas al comparar antes y después del uso de la pomada para la prueba de IVA (p = 0,15), la prueba de Schiller (p = 0,15), los cambios celulares (p = 0,47) y el análisis microbiológico (p = 0,89) mediante citología. Tras el uso, no se observó ninguna reacción adversa en la muestra estudiada.

**Conclusiones:**

Con base en los resultados identificados en este estudio, se identificó que el producto es seguro y no promueve eventos adversos. Con relación a la eficacia del producto en lesiones cervicales uterinas, es necesario continuar el estudio en fase II. Registro de Estudios Clínicos Brasileños: UTN: U1111-1218-2820.

## INTRODUCTION

Intraepithelial lesions of the cervix are localized and identifiable lesions that have a high risk of developing cancer that can be treated and eradicated when previously identified^(1,2)^. Currently, treatment options for these lesions are related to the complexity level, with the need for biopsy and histopathological examination. Depending on the results of the biopsy, a conization process may be indicated, in which the transformation zone and part of the endocervical canal of the cervix are removed^(2)^. Cervical cancer has been considered the fourth most common type of cancer, encompassing around 570,000 cases and 311,000 deaths in 2018. The estimated incidence rate by country has been varying between 2-75/100,000 women. In Africa regions, it has been identified as the type of cancer that mostly affects the female population, in which approximately 6.5% of women develop it before 75 years old^(3)^.

There are no reports in care protocols including the administration of drugs for oral or topical use, but there are clinical exams with possible surgical intervention, Although relatively effective, these practices may have some steps suppressed through the insertion of medications, avoiding the acid images or surgical intervention, consequently avoiding patients from traumatic processes, and possibly saving financial resources by the public health institutions^(4)^. Thus, the use of medicinal plants with proven biological activity and pre-clinical tests already carried out is an alternative drug administration with low cost and high effectiveness, which is already included in the RENISUS list^(5)^.

The *Stryphnodendron Adstringens* (Mart.) *Coville* is a medicinal plant, popularly known as *barbatimão*, *barba-de-timão* or *casca-da-mocidade*, typical of the *Cerrado* region and widely distributed throughout the Brazilian territory^(6)^. It has a high concentration of tannins (around 20% to 50%), and together with other chemical components, such as alkaloids, protease inhibitors (such as trypsin), flavonoids, and steroids, are responsible for promoting this species unique properties for the application in the medicinal area^(7)^. Its ability to form water-insoluble complexes with proteins provides biological activities such as astringent, healing, antifungal, anti-inflammatory, antimycotic and antiseptic effect, making it an extremely attractive substance for the pharmaceutical industry and popular use^(8,9)^.

Some in vitro pharmacological tests could provide evidence of the antibacterial, antifungal, antiparasitic, antimutagenic, antiviral, and enzymatic inhibition activity of barbatimão^(10)^. The healing, antiparasitic, anti-inflammatory, antinociceptive, gastroprotective, and antigenotoxic activities have also been proven through pharmacological tests in vivo and ex vivo^(11,12)^. When the ointment was used in fractions of 1% of the dry acetonic extract from the bark of the stems of *S. adstringens* with ethyl acetate, we observed favorable effects on the re-epithelialization of skin lesions in 4, 7, and 10 days of treatment, stimulating cell proliferation, and strengthening the evidence regarding the healing activity of Barbatimão^(13)^. Heavy metals such as silver have been used for their bactericidal and bacteriostatic properties^(14)^, as well anti-inflammatory, antimicrobial, antifungal and antiviral properties^(15,16)^. The use of AgNPs through the cell wall, thereby damaging the DNA and causing cell death in microorganisms^(17)^. The use of silver nanoparticles has been highlighted in the literature for promoting the significant increase in the induction of apoptosis and decrease in factor levels alpha tumor necrosis and Interleukin-8, as well as increased Interleukin-4, epidermal growth factor, keratinocyte growth factor and levels of keratinocyte growth factor type 2^(18,19)^.

Thus, adding the *S. adstringens* phytotherapy, popularly known as Barbatimão as an alternative treatment in the treatment of uterine lesions in women with cervical lesions needs to be investigated in the populations. Also, associations such as nanoparticles should be encouraged to investigate new methods for the effectiveness of herbal medicine. Therefore, this study aimed to investigate the safety of a new topical treatment alternative for *Stryphnodendron Adstringens* (Mart.) *Coville* (Barbatimão) both with and without the addition of silver nanoparticles and in cervical lesions.

## METHODS

### Study Design and Research Participants

We carried out a prospective, randomized, double-blind, phase 1, experimental trial, including 43 women assisted by the project’s researchers. This study is a pilot investigation to examine the feasibility of a new topical treatment alternative for cervical lesions with silver nanoparticles and *Stryphnodendron Adstringens* (Mart.) *Coville* (Barbatimão) in cervical lesions. We included women over 18 years old, clinically healthy, with diagnosis of the uterine cervix without lesions, after screening with the Schiller test and visual inspection with acetic acid (VIA) and who consented to participate voluntarily in the study. The study excluded women who were pregnant or puerperal or who intended to become pregnant, who were breastfeeding, who were hypersensitive or allergic to the compounds of the products under investigation, and who were being treated with another product under investigation. The patients were randomized into two distinct groups: Group A with 23 women who used Barbatimão gynecological ointment with silver nanoparticles and Group B with 20 women who used Barbatimão gynecological ointment without silver nanoparticles. These groups used the ointment for 7 consecutive weeks. We performed anamnesis, physical examination, specular examination with the visual inspection tests VIA and Schiller in the first gynecological consultation. Women who met the inclusion criteria were instructed and directed to perform microbiological, hematological, and biochemical tests. In the second week, we evaluated the results of the exams and recorded them in the medical record.

### Ointment Preparation

The preparation of the ointment followed the methodology described in the patent entitled “Pharmaceutical composition for the treatment of HPV infections using barbatimão extracts” number US 9,023,405 B2, granted in the United States via an application for Patent Cooperation Treaty (PCT) and by the inventors Luiz Carlos Caetano, Manoel Álvaro de Freitas Lins Neto, Pedro Accioly de Sá Paixoto Neto and Zenaldo Porfírio da Silva^(20)^. A compounding pharmacy carried out the production and coding of the ointment following all the criteria determined by the National Health Surveillance Agency (ANVISA). The ointment formulation was composed of 20g of dry alcoholic extract of *Stryphnodendron Adstringens* (Mart.) *Coville*, 80g of anionic cream (Lanette) and 0.1g of silver nanoparticles (AgNPs), with or without the addition of AgNPs. After preparation, they were stored in a refrigerator at 4°C. The concentrations regarding the use of the herbal medicine were considered using what was described in the patent titred pharmaceutical composition for the treatment of HPV infections using barbatimão extracts (https://patentscope.wipo.int/search/en/detail.jsf?docId = WO2012000070), in which was made a phytochemical, toxicological, pharmacological and clinical evaluation of alcoholic and hydroalcoholic aqueous extracts. Thus, we consider the concentration highlighted in the present cited patent.

### Follow-Up During the Use of the Ointment

The patients were monitored daily from the beginning of the use of the ointment through telephone calls guided by structured questions about possible adverse effects or difficulties in use. In addition, the patients underwent weekly consultations for assessment of the uterine cervix and safety of use. Upon return, all patients presented the applicators used, properly stored in disposable plastic bags, for control and verification of compliance with the prescription. At each consultation, a kit containing an ointment, leaflet, gynecological applicators, daily intimate protectors and plastic bags was distributed. Due to the nature of the intervention, statistics, researchers and patients were blinded to the treatment allocation. Both patient and operator blinding was performed and the control method was using a sealed envelope with specific codes. The codes were kept by a third party, not involved in these processes. Therefore, both the operator and the patient were unaware of the objectives of the study. The adverse events monitored were: bleeding, vaginal dryness, pain in the lower abdomen, burning and itching. All observations were performed by 3 different evaluators, including the return for gynecological evaluation.

### Data Collect

Data were collected between 2018 and 2020. We used visual methods to identify possible changes in the cervix such as the Visual Inspection with Acetic Acid test, Schiller test, and cervical digital photography, oncotic cytology in liquid medium and analysis of the vaginal microbiota. All exams and tests were performed before and after the use of herbal medicine. In addition to these parameters, we evaluated the women included in the study through consultations consisting of anamnesis, general physical examination, and gynecological examination. The following characteristics of the mucosa of the cervix were observed: color, size, presence of tumors, characteristics of the vaginal content (abnormal or physiological), presence of blood and its origin and lesions or color changes in the mucosa.

### Ethics Aspects

The research followed all the recommendations of Resolution nº. 466/2012 of the National Health Council (CNS) and was approved by the National Research Ethics Committee - CONEP - in 2017, under the number 2.114.908 (CAAE: 58185116.6.0000.5013). To start the assessments, the patients were previously approached for research clarifications prior to the request for signature of the Informed Consent Form. The assessments were started only upon authorization.

### Statistical Analysis

We estimated that a sample size would ensure 80% power using a two-sided type I error rate of 0.05. The calculation using electronic calculator available at http://www.lee.dante.br/pesquisa/amostragem/amostra.html. We used the Statistical Package for the Social Sciences - SPSS version 22.0 software to perform descriptive statistical analyzes frequency, percentage, mean, standard deviation, standard error, and 95% confidence interval and paired and independent evaluation through the results with Wilcoxon test and McNemar tests. Kruskal-Wallis test and Kolmogorov-Smirnov Z test assessed the distribution of normal means.

## RESULTS

Regarding sociodemographic characteristics, the average age of the participants was 40.2 years old. Regarding ethnicity, 60.5% (n = 26) declared to be brown and 34.9% (n = 15) white. We can also observe that 37.2% (n = 16) reported being married, 25.6% (n = 11) single and 20.9% (n = 9) in a common-law marriage. When observing the variables education and income, identify that 37.2% (n = 16) had finished high school and 48.8% (n = 21) had between 1 and less than 2 minimum. Considering the adequacy of the smear, 88.4% (n = 38) were satisfactory for cytological evaluation before the use of the ointment and 90.7% (n = 39) after use. Before use, 67.4% (n = 29) of the samples had the squamous, glandular, metaplastic epithelium represented in the sample and after use, the rate was 86% (n = 37). It was identified absence of adverse health-damaging effects and decreased inflammation (rate = 51.2%) in the cervix, before and after its use, promoting an anti-inflammatory action.

The transformation zone was represented in 76.8% (n = 33) of the samples before use and 83.7% (n = 36) after the use of herbal medicine. Among benign reactive or reparative cell changes, inflammation was the most frequent with 51.2% (n = 22) and after use, there were no cases. However, cases of inflammation, atrophy with inflammation before use were 9.3% (n = 4) and after use were 74.4% (n = 32). The most frequent findings of the vaginal microbiota were from Lactobacillus sp. in 60.5% (n = 26) of the samples both before and after use. The conclusion of the analysis was negative for malignancy in 74.4 (n = 32) of the smears before use and in 81.4% (n = 35) after use (Table 1).

**Table 1.** Results of the Pap smear in liquid medium before and after using the ointment with/without silver nanoparticles – Arapiraca, AL, Brazil, 2018.

Pap smear	Before		After	
	N	%	N	%
**Sample evaluation**				
Satisfactory	38	88.4	39	90.7
Poor	5	11.6	4	9.3
**Epithelium represented in the sample**				
Scaly glandular metaplastic	29	67.4	37	86
Scaly metaplastic	6	14	0	0
Scaly	3	7	2	4.7
Unsatisfactory	5	11.6	4	9.3
**Representativeness of the transformation zone**				
Yes	33	76.8	36	83.7
Not	5	11.6	3	7
Poor	5	11.6	4	9.3
**Benign reactive or repairing cellular changes**				
Inflammation. immature squamous metaplasia	10	23.3	7	16.3
Inflammation. atrophy with inflammation	4	9.3	32	74.4
Inflammation	22	51.2	0	0
Inflammation. nuclear activation	2	4.6	0	0
Poor	5	11.6	4	9.3
**Microbiology**				
Lactobacillus sp.	26	60.5	26	60.5
Lactobacillus sp. Coconuts and other bacilli.	4	9.3	7	16.2
Lactobacillus sp. Coconuts. Candida sp.	4	9.3	0	0
Supracytoplasmic bacilli (suggestive of Gardnerella/ Mobiluncus)	1	2.3	6	14
Poor	8	18.6	4	9.3
**Conclusion of the analysis**				
Negative for malignancy	32	74.4	35	81.4
Atypical cells of undetermined significance. Scaly: Possibly non-neoplastic (ASC-US)	6	14	4	9.3
Poor	5	11.6	4	9.3

When independently analyzing the groups with and without silver nanoparticles in the results of the Pap smear, we observed that there were no statistically significant differences in the distribution of benign reactive or reparative cell changes between the groups before (x^2^= 3,446 GL = 6 p = 0.75) or after (x^2^= 1.389 GL = 2 p = 0.49) use. The same occurred with the microbiological findings of cytology (before: x^2^= 8.133 GL = 6 p = 0.22; after: x^2^= 2.414 GL = 4 p = 0.66), the conclusion of the analysis (before: x^2^= 0.047 GL = 1 p = 0.82; after: x^2^= 0.27 GL = 1 p = 0.87) and the observation of the sample (before: x^2^= 0.213 GL = 3 p = 0.97; after: x^2^= 4.686 GL = 4 p = 0.32). Regarding the epithelia represented in the sample, a statistically significant relationship was found before use (x^2^= 3.848 GL = 1 p = 0.05) and without significance after use (x^2^= 0.024 GL = 1 p = 0.87) (Table 2).

Through the culture of vaginal secretion, we observed the presence of microorganisms in 88.4% (n = 38) of the samples analyzed before use and in 90.7% (n = 39) after use. Regarding the pathogenicity of the microorganisms identified before and after use, the frequencies were 16.3% (n = 7) and 34.9% (n = 15), respectively. Lactobacillus sp. were present in 55.8% (N = 24) of the samples before use and in 48.8% (n = 21) after use. Gardnerella vaginallis and Candida were also identified in 7% (n = 3) of the cases before use and in 16.3% (n = 7) after use. Among the microorganisms found, 97.7% (n = 42) did not show resistance to antibiotics before and 93% (n = 43) after using the ointment (Table 3).

**Table 2.** Comparison between Pap smear before and after the use of the ointment with/without silver nanoparticles – Arapiraca, AL, Brazil, 2018.

Pap smear	Before								After							
	Barbatimão ointment					X^2^	GL	p^1^	Barbatimão ointment					X^2^	GL	p^1a^
	No silver nanoparticles		With silver nanoparticles						No silver nanoparticles		With silver nanoparticles					
	N^b^	%	N^b^	%	Total				N^b^	%	N^b^	%	Total			
Epithelia represented in the sample					34	3.848	1	**0.05**					41	0.024	1	0.87
Scaly. glandular. metaplastic	17	50.0	11	32.4	28				20	48.8	17	41.5	37			
Scaly. metaplastic	1	2.9	5	14.7	6				–	–	–	–				
Scaly	–	–	–	–	0				2	4.9	2	4.9	4			
Benign reactive or repairing cellular changes					37	3.446	6	0.75					42	1.389	2	0.49
Inflammation. metaplasia. scaly immature	5	13.5	2	5.4	7				2	4.8	4	9.5	6			
Inflammation. atrophy with inflammation	3	8.1	2	5.4	5				19	45.2	14	33.3	33			
Inflammation	11	29.7	12	32.4	22				2	4.8	1	2.4	3			
Inflammation. nuclear activation	1	2.7	1	2.7	2				–	–	–	–	0			
Microbiology					34	8.133	6	0.22					39	2.414	4	0.66
Lactobacillus sp.	11	32.4	10	29.4	21				11	28.2	13	33.3	24			
Lactobacillus sp. Coconuts and other bacilli.	3	8.8	5	14.6	8				5	11.9	3	7.7	8			
Lactobacillus sp. Coconuts. Candida sp.	3	8.8	1	2.9	4				–	–	–	–	0			
Supracytoplasmic bacilli (suggestive of Gardnerella/Mobiluncus)	1	2.9	0	0	1				5	12.8	2	5.1	7			
Conclusion of the analysis					37	0.047	1	0.82					39	0.27	1	0.87
Negative for malignancy	17	45.9	14	37.8	31				19	48.7	16	41.0	35			
Atypical cells of undetermined significance. Scaly: Possibly non-neoplastic (ASC-US)	3	8.1	3	8.1	6				2	5.1	2	5.1	4			
Sample observation					30	0.213	3	0.97					43	4.686	4	0.32
Nonspecific chronic cervicitis with squamous metaplasia	9	30.0	6	20.0	15				6	14.0	10	23.3	16			
Chronic bacterial cervicitis with squamous metaplasia and nuclear activation	4	13.3	3	10.0	7				6	14.0	4	9.3	10			
Chronic mycotic cervicitis with squamous metaplasia	1	3.3	1	3.3	2				3	7.0	2	4.7	5			
Suggested colposcopy with directed biopsy.	3	10.0	3	10.0	6				8	18.6	3	7.0	11			
Poor	–	–	–	–	0				0	0.0	1	2.3	1			

^a^ Significance for the Mc Nemar test. b Used binomial distribution. *X* ^2^ Chi-square test value. GL Degree of Freedom. *p* ^1^ Significance for the Chi-square test.

**Table 3.** Independent statistical analysis and dependent on the results of the vaginal secretion culture between the groups with or without silver nanoparticles before and after the use of gynecological ointment – Arapiraca, AL, Brazil, 2018.

Culture of vaginal secretion	Before								After								
	Barbatimão ointment					**X** ^2^	GL	**p** ^1^	Barbatimão ointment					**X** ^2^	GL	**p** ^1^	**p** ^a^
	No silver nanoparticles		With silver nanoparticles						No silver nanoparticles		With silver nanoparticles						
	N	%	N	%	Total N				N	%	N	%	Total N				
**Presence of Microorganism**					43	0.414	1	0.52					43	0.022	1	0.883	1.00^c^
Absent	2	4.65	3	6.97	5				2	4.65	2	4.65	4				
Present	21	48.8	17	39.5	38				21	48.8	18	41.8	39				
**Pathogenicity**					43	1.082	1	0.29					43	0.393	1	0.531	0.02^b^
Normal Microbiota	18	41.8	18	41.8	36				14	32.5	14	32.5	28				
Pathogenic	5	11.6	2	4.65	7				9	20.9	6	13.9	15				
**Aerobic/Anaerobic**					43	0	0	0					43	0.890	1	0.345	1.00^b^
Optional anaerobic	23	53.4	20	46.5	43				22	51.1	20	46.5	42				
Anaerobic	0	0.0	0	0.0	0				1	2.33	0	0.0	1				
**Antibiotic resistance**					43	0.890	1	0.34					43	0.527	1	0.468	0.62^b^
No	22	51.1	20	46.5	42				22	51.1	18	41.8	40				
Yes	1	2.33	0	0.0	1				1	2.33	2	4.65	3				
**Gram positive/Gram negative**					43	0.820	1	0.36					43	0.890	1	0.345	0.37^b^
Positive	20	46.5	19	44.1	39				22	51.1	20	46.5	42				
Variable	3	6.71	1	2.33	4				1	2.33	0	0.0	1				
**Search for Trichomonas vaginallis**					43	0	0	0					43	0	0	0	
Absent	23	53.4	20	46.5	43				23	53.4	20	46.5	43				
Present	0	0.0	0	0.0	0				0	0.0	0	0.0	0				
**Search for Gardnerella vaginallis**					43	0.225	1	0.63					43	1.082	1	0.298	0.12^b^
Absent	21	48.8	19	44.1	40				18	41.8	18	41.8	36				
Present	2	4.65	1	2.33	3				5	11.6	2	4.65	7				
**Search for Monilia**					43	0.096	1	0.75					43	0.045	1	0.832	0.68^b^
Absent	20	46.8	18	41.8	38				19	44.1	17	39.8	36				
Present	3	6.70	2	4.65	5				4	9.30	3	6.70	7				

^a^ Significance for the Mc Nemar test. b Used binomial distribution. *X* ^2^ Chi-square test value. GL Degree of Freedom. *p* ^1^ Significance for the Chi-square test.

## DISCUSSION

This study evaluated the safety of Barbatimão gynecological ointment, comparing one group with and one without silver nanoparticles in a healthy cervix. To release an herbal medicine for use and sale, it needs some phases such as phase I, performed with groups of 20 to 100 healthy individuals^(20)^. The use of herbal medicine was performed as an ointment and in one of the evaluation options, the ointment contained silver nanoparticles. It was applied in different ways, with the intention that the final product may constitute a dressing. One of the ways to be assessed includes coverage of the cervix^(18)^. However, it is intended that this approach can prove the pharmacological potential of Barbatimão ointment with metal nanoparticles, while hopefully avoiding the numerous acid-imaging procedures and conization of cervical cancer cases^(19)^.

The main results obtained in this study were the absence of adverse health-damaging effects and decreased inflammation in the cervix. We observed that before its use the inflammation rate was 51.2%, while after its use, there were no cases, promoting an anti-inflammatory action. When the groups were analyzed independently, there were no statistically significant differences between the groups with or without silver nanoparticles before or after using it. The tannins present in the Barbatimão act in the healing process and reduction of inflammation through the protective layer formed by the tannin-protein complex on the injured skin or mucosa^(21)^.

The healing, antibacterial, anti-inflammatory, antifungal, antiparasitic and antiviral activities of Barbatimão have been reported through in vitro, in vivo, and popular use studies^(10,22)^. The effects and properties of *Stryphnodendron Adstringens* and beach extract have been tested for the anti-inflammatory effect of Barbatimão and other species on the production of tumor necrosis factor-alpha (TNF-𝛼), as well as its anti-arthritis activity in mice found a relationship with a decrease leukocyte migration to the inflammatory site^(23)^.

Through the vaginal secretion culture, we could determine the presence of microorganisms, pathogenic or not, of the vaginal microbiota. The majority were found in *Lactobacillus sp*. (55.8%), natural microorganisms from the vaginal microbiota that do not have a risk to women’s health. However, *Staphylococcus*, *Enterobacter*, *Streptococcus*, and *Escherichia coli* species have also been found, although few. Women who presented pathogenic microorganisms after using the ointment were referred for treatment with medical monitoring.

In microbiological tests the broad antimicrobial spectrum of the hydroethanolic extracts of Barbatimão was confirmed, and they observed the activity of all extracts against *Candida, Staphylococcus aureus*, and *P. aeruginosa* ^(24)^. However, in this study, we did not identify antimicrobial activity since the pathogenic microorganisms identified before use were 16.3% and after use 34.9%. Concentration of dry extract of Barbatimão is adequate to inhibit the development of *Staphylococcus epidermidis* and *E. coli* would be 75mg/mL, while for S. aureus 50mg/mL would be enough. In this study, we used a lower concentration, which may justify the absence of antimicrobial action^(25)^.

The toxic effect of Barbatimão was tested in a study in rats, where it was demonstrated that the concentrations deemed toxic were 800 mg/kg and 1600 mg/kg during 30 days of use^(24)^. These doses are much higher than those used in this study. The clinical evaluation parameters were performed through visual inspection tests with acetic acid and Schiller, in addition to weekly monitoring, with no complaints related to use were reported by the patients. No similar studies were found evaluating the safety of Barbatimão ointment in the cervix. Thus, this study may assist in conducting future research, considering that nature is an important source of molecules of pharmaceutical utility, and its natural products can be used in their natural formula or pharmaceutical formulations, better known as herbal medicines^(25)^. We believe that the major limitation of our study involves the issue of having performed the safety test directly in a population.

## CONCLUSIONS

The results of this study conclude that the Barbatimão gynecological ointment with and without silver nanoparticles does not have a risk to human health since no adverse effects were observed or harmful to human health. The ointment tested here has anti-inflammatory activity, proven through the result of oncotic cytology in a liquid medium and visual inspection tests of the cervix before and after use. Therefore, the results in this study demonstrated that the proposed topical formulation is safe for use in the cervix during the weeks of use investigated.

For the purposes of the development of new research, new clinical trials must be carried out applied to different clinical stages of cervical cancer and investigation of the prognosis in the treatment of the disease. With this research, it is expected that the product will contribute to reduce the number of injuries that evolve into more serious forms, preventing women from being subjected to more invasive and painful procedures, in addition to enabling a reduction in public spending.
